# The Role of the Transcription Factor Nrf2 in Alzheimer’s Disease: Therapeutic Opportunities

**DOI:** 10.3390/biom13030549

**Published:** 2023-03-17

**Authors:** Laura Maria De Plano, Giovanna Calabrese, Maria Giovanna Rizzo, Salvatore Oddo, Antonella Caccamo

**Affiliations:** Department of Chemical, Biological, Pharmaceutical and Environmental Sciences, University of Messina, Viale F. Stagno d’Alcontres 31, 98168 Messina, Italy

**Keywords:** APP, tau, neurodegeneration, brain aging, oxidative stress, neuroinflammation

## Abstract

Alzheimer’s disease (AD) is a common neurodegenerative disorder that affects the elderly. One of the key features of AD is the accumulation of reactive oxygen species (ROS), which leads to an overall increase in oxidative damage. The nuclear factor (erythroid-derived 2)-like 2 (Nrf2) is a master regulator of the antioxidant response in cells. Under low ROS levels, Nrf2 is kept in the cytoplasm. However, an increase in ROS production leads to a translocation of Nrf2 into the nucleus, where it activates the transcription of several genes involved in the cells’ antioxidant response. Additionally, Nrf2 activation increases autophagy function. However, in AD, the accumulation of Aβ and tau reduces Nrf2 levels, decreasing the antioxidant response. The reduced Nrf2 levels contribute to the further accumulation of Aβ and tau by impairing their autophagy-mediated turnover. In this review, we discuss the overwhelming evidence indicating that genetic or pharmacological activation of Nrf2 is as a potential approach to mitigate AD pathology.

## 1. Introduction

Alzheimer’s disease (AD) is the most diffuse form of dementia, and it is estimated that over 55 million people worldwide may have AD [1,2]. A threefold increase in prevalence is expected by 2050 if no cure or treatment is found within the next few years [2]. The majority of AD cases are sporadic and normally have an age of onset higher than 65 years of age. The causes of sporadic AD are unknown, even though several genetic and environmental risk factors have been identified [3]. About 1% of all cases of AD are genetic and are associated with an average age of onset in the mid-40s. These genetic cases are due to autosomal dominant mutations in one of three genes: the amyloid precursor protein (APP), presenilin 1 (PS1) and presenilin 2 (PS2). All three genes are involved in the production of a small peptide known as amyloid-β (Aβ).

Memory loss, specifically episodic memory, is one of the earliest clinical manifestations of AD [4]. However, as the disease progresses cognitive decline becomes more severe and affects multiple cognitive domains; indeed, deficits in semantic memory, attention, executive functions, and language are very common in people with severe AD [5]. Eventually, patients become bedridden and succumb to comorbidities associated with the disease.

The major hallmarks of AD are extracellular plaques and intracellular neurofibrillary tangles (NFTs) [1]. Plaques are extracellular aggregates mainly made of Aβ, a small peptide that derives from the pathological processing of APP. Physiologically, APP is cleaved by metalloproteases (e.g., A disintegrin and metalloproteinase 10) right in the middle of the Aβ sequence, thereby precluding Aβ formation. In AD, APP is first cleared by BACE1 (β-site amyloid precursor protein (APP) cleaving enzyme 1) right at the beginning of the Aβ sequence. BACE1 cleavage generates a C-terminal fragment of APP known as C99, which is further cleaved by the γ-secretase complex to generate Aβ. PS1 is the main catalytic subunit of the γ-secretase complex [1]. Once generated, Aβ self-aggregates to form toxic Aβ oligomers and eventually extracellular Aβ plaques.

Tangles are made of hyperphosphorylated tau, a microtubule-binding protein [1]. Physiologically, tau binds to microtubules stabilizing their structure. However, pathological tau is hyperphosphorylated and binds poorly to microtubules, causing several alterations in neuronal function [6]. As a result, acute or chronic reduction in tau levels leads to synaptic and cognitive deficits [7,8]. The causes underlying tau accumulation and neurofibrillary tangle formation in AD remain unknown.

The accumulation of plaques and tangles promotes local inflammatory response and contributes to neurotoxicity [1]. Indeed, neuropathological examinations of postmortem human AD brains show a high degree of local inflammation, especially surrounding extracellular Aβ plaques. An established way by which Aβ and tau accumulation increases overall neuroinflammation in AD is by activating microglia and astrocytes (reviewed in [9]). Microglia are the innate immune cells of the central nervous system. Astrocytes, specialized glial cells, are activated by pathological insults and may phagocytose Aβ. The activation of microglia is initially thought to be beneficial for the brain as microglia may also phagocytose Aβ. However, chronic microglia activation, as in AD, leads to the secretion of cytokines which may contribute to exacerbating the neuropathological phenotype by increasing tau phosphorylation and by contributing to neuronal death [9].

Another feature of AD is mitochondrial dysfunction and oxidative stress, which are linked to the excessive production of reactive oxygen species (ROS). The accumulation of ROS in neurons causes damage to mitochondria, which, in turn, leads to more production of ROS and eventually neuronal death [10]. Several mechanisms have been proposed to explain the formation and accumulation of ROS in AD. For example, a proposed mechanism suggests that chronic activation of microglia in the brain of AD patients activates nicotinamide adenine dinucleotide phosphate oxidase, which impairs mitochondrial function leading to ROS production [11]. Another independent pathway that may increase ROS production in AD is mediated by mutated *PS1* and *PS2*. Using complementary models, Muller and colleagues showed that mutations in these two genes augment the activity of the inositol trisphosphate receptor leading to an increase in intracellular calcium concentration, which causes oxidative damage and ROS production [12]. Additionally, multiple laboratories have shown a direct interaction between Aβ and mitochondria. To this end, Aβ has been found to interact and block the voltage-dependent ion channel on the outer membrane and to directly accumulate inside mitochondria. Both these events lead to mitochondrial dysfunction and the accumulation of ROS [13].

Under physiological conditions, an increase in ROS stimulates cells to produce a series of antioxidant molecules to prevent oxidative damage [10]. The nuclear factor (erythroid-derived 2)-like 2 (Nrf2) is a transcription factor, activated by oxidative stress, that regulates the expression of the antioxidant response element-dependent genes, which are needed to reduce oxidative stress. However, in AD, Nrf2 levels are decreased, thereby hampering the activation of the neuronal antioxidant response pathways [14,15]. In this review, we evaluate the current literature describing the role of Nrf2 in Aβ and tau accumulation and the cognitive deficits associated with AD.

## 2. Nrf2 Structure and Function

**Figure 1:**
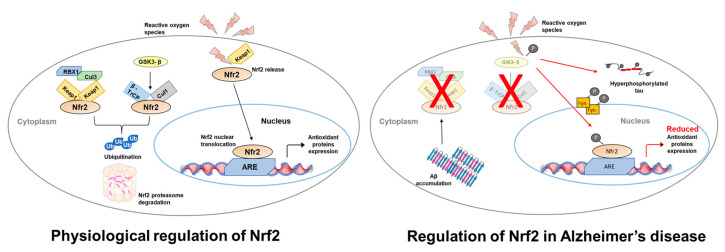
Schematic representation of Nrf2 regulation in neurons. Under physiological conditions Nrf2 signaling is regulated in two ways: (i) Keap 1 sequesters Nrf2, which leads to Nrf2-ubiquitination through the formation of the Keap1–Cul3-Rbx1 complex. Nrf2 is then targeted to and degraded by the proteasome; (ii) GSK-3β phosphorylates Nrf2, which then binds to an E3 ligase and β-TrCP. This allows the ubiquitin-proteasome degradation of Nrf2. High ROS levels lead to positive regulation of the Nrf2 pathway by releasing Nrf2 from Keap1. Nrf2 is then free to translocate into the nucleus where it binds ARE gene sequences activating their expression. In AD, Aβ_42_ accumulation (i) increases ROS production and (ii) blocks the regulation of Nrf2 by stabilizing the interaction between Keap1 and Nrf2. This ultimately leads to a reduced expression of the oxidative stress response genes, which further leads to an increase in ROS levels. Moreover, GSK-3β, which is involved in tau phosphorylation, contributes to Nrf2 degradation by proteasome via a Fyn-mediated mechanism.

Nrf2 is a transcription factor that, in humans, is encoded by the *NFE2L2* gene. It is a member of the basic leucine zipper (bZIP) transcription factors called ‘cap n collar’ and recognizes and binds to the antioxidant response element (ARE) present in its target genes [16]. Its main function is to protect cells from oxidative stress, thus maintaining the cells’ redox homeostasis. Normally, Nrf2 is found in the cytosol where it forms a complex with Kelch-like ECH-associated protein 1 (Keap1), which keeps Nrf2 in an inactive state. Under homeostatic conditions, Keap1 mediates the ubiquitination of Nrf2 through its interaction with cullin-dependent E3 ubiquitin ligase, which eventually leads to the degradation of Nrf2 and the suppression of its activity [16,17,18]. When cells undergo oxidative stress, the cysteine sulfhydryl groups of Keap1 are modified resulting in a detachment of Keap1 from Nrf2, which, in turn, is transported into the nucleus [17,18] (Figure 1). Consistent with these observations, the steady-state levels of Nrf2 are increased in Keap1 knockout mice, leading to a constitutively active Nrf2 signaling pathway [19,20].

Nuclear translocation is also regulated by phosphorylation of Nrf2 at serine 40 by several kinases, such as phosphoinositide-3 kinase/protein kinase B (PI3K/AKT), glycogen synthase kinase 3 (GSK-3β) and protein kinase C (PKC) [21,22]. For example, phosphorylation of Nrf2 by PKC is needed for its detachment from Keap1 [23]. In contrast, phosphorylation of Nrf2 at different amino acid residues may have opposite effects on Nrf2 activation. Indeed, it has been reported that phosphorylation of Nrf2 by mitogen-activated protein kinases stabilizes its interaction with Keap1, thereby reducing Nrf2 translocation to the nucleus [24]. In the nucleus, over 200 ARE-containing genes can be activated by Nrf2; these include NADPH, superoxide dismutase, catalase, gamma-glutamylcysteine synthetase, heme oxygenase-1, glutathione reductase and S-transferase, thioredoxin reductase, peroxiredoxins and some heat shock proteins [25,26,27].

Given the importance of Nrf2 in protecting cells from oxidative stress, its levels in cells are tightly controlled by a balance between production and degradation. In a study by Kwak and colleagues, it was reported that Nrf2 can regulate its own transcription through ARE-like sequences found within its promoter region [28]. Its degradation is regulated by a ubiquitin-proteosome Keap1-dependent pathway in the cytosol and a Keap1-independent mechanism in the nucleus [29,30]. Another way that cells use to regulate Nrf2 signaling is by increasing its stabilization. For example, following oxidative stress, the Nrf2 half-life almost doubles [31]. Overall, both overactivation and underactivation of Nrf2 signaling have been linked to several diseases, such as cancer, AD, and rheumatoid arthritis [14,32,33,34].

**Figure 2:**
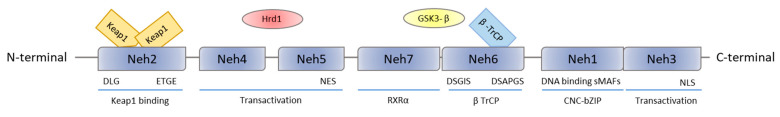
Nrf2 protein structure. Nrf2 has seven Neh domains: Neh1 domain containing bZip regions. ETGE and DLG motifs, on Neh2, are the sequences through which Keap1 binds to Nrf2. The Neh3 domain contains nuclear localization sequences. Neh4 and Neh5 are transactivation domains and are bound by a ubiquitin ligase, such as Hrd1. On Neh6, DSGIS and DSAPGS motifs are used by βTrCP ubiquitin ligase. Finally, through the Neh7 domain, Nrf2 interacts with RXRα protein, inducing Nrf2 repression.

Structurally, Nrf2 is composed of seven Nrf2-ECH homology (Neh) domains (Figure 2). Neh1 is the CNC-bZIP domain needed for DNA binding. The motifs DLG and ETGE in Neh2 are responsible for the interaction of Nrf2 with Keap1, while, the lysine residues are important for Nrf2 ubiquitination through the formation of the Keap1–Cul3-Rbx1 complex, thereby contributing to Nrf2 degradation by the proteasome [35]. The DSGIS and DSAPGS motifs in Neh6 mediate the interaction with E3 ligase adapter β-TrCP after GSK-3β-mediated phosphorylation of the DSGIS motif [22]. The Neh7 domain is involved in the repression of Nrf2 transcriptional activity by the retinoid X receptor α through a physical association between these two proteins and Nrf2. The Neh 3, 4, and 5 domains are important for transactivation [22].

## 3. The Role of Nrf2 in the Cognitive Deficits Associated with AD

In the brain, *NFE2L2* is highly expressed in astrocytes and microglia and less in neurons [25]. In astrocytes Nrf2 increases the expression levels of glutathione-synthesis-related genes and enhances the synthesis of glutathione (GSH), which is then transported to neurons where it protects them from oxidative damage [36,37,38]. In microglia, Nrf2 modulates microglia dynamics by reducing cyclooxygenase 2 (COX2), nitric oxide synthase 2 (NOS2), IL-6, and tumor necrosis factor (TNF) production and by increasing the levels of several anti-inflammatory markers [39].

Nrf2 levels and activity are reduced in the brain of aging people and the CA1 of AD patients [15,40,41]. Specifically, Ramsey and colleagues showed that in the hippocampus of AD patients, Nrf2 is mainly found in the cytoplasm, as indicated by immunohistochemistry and confocal imaging analyses [15]. The same group confirmed these data by cellular fractionation and western blot and reported that, in the nuclear fraction of hippocampal AD neurons, the steady-state levels of Nrf2 were significantly reduced compared to those obtained from age-matched healthy controls [15]. Interestingly, Gureev and colleagues showed an age-dependent decrease in *NFE2L2* mRNA levels in the cortex of wild-type mice [40], suggesting an alternative link (i.e., independent of the accumulation of Aβ and tau) between aging and onset of oxidative damage in AD. Consistent with these observations, Bahn and colleagues reported an association between Nrf2 and BACE1. They showed that reducing Nrf2 levels increases BACE1 activity and therefore Aβ production, an event independent of oxidative stress [41].

Further strengthening the link between AD and Nrf2 is the finding that a specific haplotype of the *NFE2L2* gene is linked to the progression of AD [42]. Several studies have demonstrated that the abnormal regulation of Nrf2 in AD brains is mainly associated with the accumulation of misfolded proteins and the increase in oxidative stress [43,44]. Particularly, the accumulation of Aβ leads to the formation of free radicals by activating NADPH oxidase and by increasing ROS production [13,45,46]. Similarly, an increase in tau phosphorylation and aggregation increases ROS production [45].

In APP/PS1 mice, a common mouse model of AD, the levels of Nrf2 and its target genes are significantly reduced [43]. Specifically, the authors showed an inverse correlation between the accumulation of Aβ and *NFE2L2* mRNA and protein levels in the CA3 region of the hippocampus. In this regard, in 3-month-old mice, which have practically no Aβ pathology, the levels of Nrf2 were similar between transgenic and non-transgenic mice. However, as the mice aged, the levels of Aβ significantly increased while the levels of Nrf2 significantly decreased. Finally, the authors also showed that the decrease in Nrf2 corresponded to a reduced expression of three known targets of the Nrf2 pathway, NQO1, GCLC and GCLM [43]. Consistent with these observations, Rojo and colleagues conducted a microarray analysis in Nrf2 knockout animals and found that several functional pathways that are known to be altered in AD brains were also altered in these mice [47], further implicating Nrf2 as a possible player in the pathogenesis of AD.

Several groups have reported that genetically reducing *NFE2L2* in animal models increases oxidative stress and inflammation, replicates gene alteration in gene expression typical of AD brains and exacerbates cognitive deficits [47,48,49]. Along the same lines, downregulating *NFE2L2* expression, using an Nrf2-shRNA-lentivirus, in the hippocampus of young senescence-accelerated mouse prone 8 altered synaptic plasticity and accelerated cognitive impairments [50]. We removed both copies of the *NFE2L2* gene from APP/PS1 mice by breeding them with *NFE2L2* knockout mice [49]. The lack of *NFE2L2* in 12-month-old APP/PS1 mice exacerbated cognitive deficits as we found a worsening of spatial learning and memory, working memory, and associative memory [49]. The deterioration was linked to a reduction in HO-1 levels and an increase in Aβ and interferon-gamma levels [49]. Consistent with these observations, other groups have shown that reducing the levels of *NFE2L2* also increases brain inflammation [51].

Together, these and other data have suggested that increasing Nrf2 function might be a novel venue to mitigate AD pathology. Indeed, increasing Nrf2 activity in animal models ameliorates AD-like pathology and the associated cognitive deficits. For example, genetically increasing *NFE2L2* levels in AppNL-G-F/NL-G-F knock-in mice reduced inflammation and oxidative stress and improved cognition [36]. In a complementary experiment, Kanninen and colleagues used a lentiviral vector to increase the expression of *NFE2L2* levels in the hippocampus of APP/PS1 mice. They reported that *NFE2L2*-gene transfer was associated with a reduction in astrocytic, but not microglial, activation and an improvement in spatial learning and memory [52]. Consistent with the genetic approaches, several small molecules have been shown to increase Nrf2 function and decrease Aβ and tau accumulation e.g., [53,54,55].

While the mechanisms underlying the decrease in Nrf2 levels and activity in AD remain elusive, a strong link has emerged between Aβ accumulation and Nrf2 reduction. In this regard, Aβ deposition increases the levels of Nrf2 suppressors (Keap1 and GSK-3β), thereby leading to a reduction in Nrf2 signaling [56]. Along the same lines, others have reported that the initial accumulation of Aβ leads to an increase in both Nrf2 and Keap1 levels. The increase in Nrf2 levels is a protective initial response to Aβ accumulation. However, the continued upregulation of Keap1 does eventually inhibit Nrf2 contributing to the progression of the disease [15,57]. The reduction in Nrf2 may increase BACE1 activity, thereby further increasing Aβ production and Nrf2 reduction [41], creating a vicious cycle.

## 4. Regulation of *NFE2L2* mRNA Translation in AD

In addition to regulating the activation of the Nrf2 pathway by its interaction with Keap1, cells control the total levels of *NFE2L2* expression by selective micro-RNAs (miRNAs), non-coding RNA involved in the regulation of gene expression and various biological processes. miRNAs interact with the target mRNA through base-pairing and induce its degradation or the inhibition of its translation. Growing evidence points to the role of miRNAs in the pathogenesis of several neurodegenerative disorders, including AD. For example, recently, it has been suggested that miRNA-143-3p, whose expression is reduced in AD brains, regulates both tau phosphorylation and Aβ production [58]. Along the same lines, miRNA-455-3p binds to the APP mRNA thereby reducing APP levels and Aβ production [59]. miRNA-298 is another miRNA that has been linked to AD as it regulates the expression of APP and BACE1 in a cell-specific manner. Indeed, increasing the expression of miRNA-298 reduces the expression of APP and BACE1 in astrocytes but not in neurons [60]. Given their role in regulating key genes involved in the pathogenesis of AD, miRNAs are increasingly becoming a therapeutic target.

Physiologically, miRNAs are also involved in the regulation of genes involved in the response to oxidative stress and neuroinflammation, including *NFE2L2*. To this end, Narasimhan and colleagues used bioinformatic analysis to identify miRNAs that might target human *NFE2L2*. Of the miRNAs identified, they empirically showed, using a neuronal-like cell line, that miRNAs 27a, 144, 142-5p and 153 directly interact with Nrf2 3′UTR leading to a reduction in Nrf2 mRNA translation and an overall decrease in the steady-state levels of Nrf2 [61]. Similarly, miRNA-28 and 340-5p directly bind to the *NFE2L2* mRNA reducing its translation [62,63]. miRNA can also positively regulate Nrf2 signaling by interacting with Keap1. For example, miR-432 directly interacts with Keap1 mRNA, thereby blocking its translation [64]. Similar outcomes have been reported for miRNA-592 [65]. The miRNA-mediated downregulation of Keap1 leads to an overall increase in Nrf2 signaling.

Some miRNAs have been directly linked to AD pathogenesis through an Nrf2-mediated mechanism. For example, miRNA142 and some of its variants are linked to inflammation and oxidative stress by Nrf2-mediated mechanisms [66]. Converging evidence indicates that the levels of miRNA142 are upregulated in the hippocampi of AD patients and APP transgenic mice (e.g., [67,68]). In addition, miRNA142 has been genetically linked to sporadic AD [69]. While the exact mechanisms linking miRNA142 to AD remain unclear, it is tempting to speculate that an increase in its levels would decrease Nrf2 mRNA translation and consequently reduce Nrf2 signaling, making cells more susceptible to oxidative stress. An elegant study by Zhao and colleagues showed that the levels of miRNA-28 positively correlated with AD status and progression. Indeed, they were the lowest in healthy controls and gradually increased in MCI and AD patients. While the authors did not comment on the possible mechanisms linking AD to miRNA-28, a careful analysis of the literature would suggest that miRNA-28 may contribute to AD progression by interacting with Nrf2 mRNA and blocking its translation. Overall, more needs to be done to elucidate the mechanistic link among miRNAs, Nrf2 and AD pathogenesis.

## 5. Autophagy, Nrf2, and Alzheimer’s Disease

Autophagy is a quality control system involved in the degradation of proteins and intracellular structures, such as mitochondria. There are three major types of autophagy: macroautophagy, microautophagy and chaperon-mediated autophagy. While the mechanisms leading to the activation of these three types of autophagy differ from each other, the ultimate result is the delivery of cargo (e.g., misfolded proteins) to the lysosome for degradation [70]. During the induction of macroautophagy (herein simply referred to as autophagy) a double-membrane cytoplasmic structure forms around the proteins/organelles to be removed. Eventually, this double membrane closes forming the autophagosome, a vesicle that will fuse with the lysosome for cargo degradation [70]. Overwhelming evidence shows that different aspects of the autophagy process are impaired in AD and related disorders. For example, in an elegant work, Reddy and colleagues showed that both mutant APP and Aβ inhibit autophagy induction in a hippocampal cell line [71]. Consistent with these observations, Nixon and colleagues showed an impairment of autophagy flux in AD. Specifically, they reported the accumulation of autophagosomes in postmortem human AD brains and mouse models of AD [72]. We also reported that the accumulation of Aβ impairs autophagy induction; this event reduces Aβ clearance, thereby further increasing its levels [73]. Consistent with this model, multiple laboratories have shown that increasing autophagy induction and/or flux ameliorates AD-like pathology in mice (reviewed in [73]).

p62 is a key protein involved in autophagy. For example, p62 binds to polyubiquitinated proteins and targets them to the autophagosome. Indeed, deficits in the autophagy process lead to p62 accumulation, which can, in turn, activate the cell stress response system [74]. Recently, it has become apparent that, in addition to autophagy, p62 can regulate other cellular functions [74]. Particularly relevant is the crosstalk between Nrf2 and p62 [75]. To this end, p62 can directly interact with Keap1, thereby precluding its interaction with Nrf2, which can then translocate into the nucleus. Nrf2, in the nucleus, stimulates the expression of many genes, including p62, effectively creating a positive feedback loop [76]. Based on these observations, it is tempting to speculate that, in AD, a deficit in autophagy leads to p62 accumulation, which in turn can activate Nrf2 signaling. However, the chronic activation of this loop and the further accumulation of p62 might further contribute to autophagy impairments and neurodegeneration. Consistent with these observations, reduced Nrf2 signaling can increase tau phosphorylation by decreasing autophagy, which, in turn, leads to a higher tau, and even Aβ [77,78,79]. In this regard, the link between autophagy and Aβ/tau is well-established (e.g., [80]).

## 6. Nrf2, Heat Shock Proteins and AD

AD is considered a misfolded protein disease [1]. Physiologically, protein folding is aided by heat shock proteins (HSPs) and, thus, it is not surprising that alteration in the function of various HSPs has been linked to AD [81]. The heat shock factor 1 (HSF1) is considered a master regulator in the HSP response. Upon accumulation of misfolded proteins, such as Aβ and tau, HSF1 leads to the activation of Hsp90 and stress-inducible protein 1 in an attempt to properly re-fold the misfolded proteins [81]. Importantly, some genes coding for HSPs contain an ARE consensus sequence and, as such, represent canonical targets for Nrf2.

Upon activation, HSF1 binds directly to Aβ oligomers to neutralize them; in contrast, knocking out the *HSF1* gene leads to apoptosis [82]. Paul and colleagues using complementary approaches indicate that Nrf2 increases the expression of HSF1 [83]. Thus, the accumulation of Aβ oligomers increases inflammation and oxidative damage leading to an increase in Nrf2 activity. Nrf2, in turn, increases the expression of HSF1, which triggers the HSP response and directly attacks Aβ oligomers [82]. However, as discussed above, the chronic presence of Aβ oligomers eventually leads to upregulation and stabilization of Keap1, thereby decreasing Nrf2 signaling. Ultimately, this would decrease the Nrf2-mediated increase in HSF1 leading to further accumulation of Aβ oligomers.

Using complementary approaches, two groups have shown a direct physical interaction between HSP90 and Nrf2 and HSP90 and Keap1 [83,84]. Notably, it has been postulated that the binding of HSP90 to Nrf2 would increase the stability and, thus, the activity of Nrf2 [84]. This is highly germane to AD as a large body of evidence indicates that HSP90 activation may decrease AD-like pathology developed by mouse models of this disease [81,85]. While the mechanism mediating this improvement remains to be established, in a recent work, Okusha and colleagues showed that one way by which Hsp90 may attempt to protect the brain from Aβ-induced damage is by activating Nrf2 [86]. Indeed, they showed that increasing the levels of Hsp90 led to higher Nrf2 phosphorylation at serine 40, which ultimately resulted in increased Nrf2 transcriptional activity.

HSP32 (also known as HO1), whose steady-state levels are increased in the hippocampus and temporal cortex of AD patients [87], appears to colocalize with plaques and tangles [88]. The increased expression of HO1 is already evident in MCI patients and, as such, is considered an early event in disease pathogenesis and a consequence of Aβ accumulation and oxidative stress [89]. Once upregulated, HO1 attempts to mitigate the oxidative damage and the overall pathological scenario by multiple mechanisms, including induction of autophagy. Notably, Nrf2 is one of the transcription factors that regulate the expression of HO1. Thus, the initial upregulation of HO1 in disease pathogenesis might be due to an early increase in Nrf2 levels; however, as we discussed above, as the disease progresses, Nrf2 levels decrease and, as such, HO1 expression might be kept high by other transcription factors.

## 7. Pharmacological Activation of the Nrf2 Pathway

A major attempt has been made to identify compounds that can increase Nrf2 signaling. For example, Rong and colleagues reported that rosmarinic acid (RosA) attenuates Aβ-induced oxidative stress by activating the Nrf2 pathway. Mechanistically, they found that RosA induces the accumulation of Nrf2 in the nucleus and stimulates the Nrf2/ARE defense system by activating the protein kinase B/GSK3β pathway [90]. Consistent with these observations, direct activation of GSK3β by lithium increases the transcription of Nrf2 target genes in a dose-dependent manner [91,92].

Carnosic acid (CA), a phenolic diterpene compound present in rosemary and sage, is another molecule used to increase Nrf2 activation [93]. Two independent mouse models of AD have been dosed with CA: J20 mice, which overexpress human APP, and 3xTg-AD mice, which overexpress human APP and tau and harbor a FAD-linked mutation in the PS1 gene [94,95,96]. In J20 mice, CA administration decreased Aβ levels and improved cognitive function [93]. In 3xTg-AD mice, CA administration decreased inflammatory markers and tau phosphorylation. The authors did not report whether CA also improved cognitive deficits in 3xTg-AD mice [93]. CA beneficial effects could be mediated by an increase in alpha-secretase activity and a subsequent decrease in Aβ production [97]. 

Interestingly, Mini-GAGR, a compound commonly used as a human food additive, crosses the blood-brain barrier and dissociates Nrf2 from Keap by increasing Ser-40 phosphorylation of Nrf2 [98]. Intranasal administration of mini-GAGR to 3xTg-AD mice reduced Aβ and tau accumulation and improved cognitive deficits [98]. Gracilin A, a sponge-derived diterpenoid compound, induced Nrf2 nuclear translocation, which, in turn, reduced Aβ_42_ and tau phosphorylation in 3xTg-AD mice. Unfortunately, under the conditions used in this work, gracillin-A did not significantly improve cognitive deficits [99]. In APP/PS1 mice, hydrogen sulfide increases Nrf2 signaling, which leads to reduced Aβ levels and improved cognitive function [100].

Nrf2 function can also be increased by forsythoside, a natural product from the phenylpropanoid glycoside group found in many plants. In male APP/PS1 mice, chronic forsythoside administration increased Nrf2 signaling and mitigated Aβ accumulation and tau phosphorylation. These changes were also associated with an improvement in cognitive function [101]. Similarly, anthocyanin, a natural pigment found in plants, has been given to APP/PS1 mice. Consistent with the forsythoside study mentioned above, anthocyanin improved AD-like pathology by increasing the Nrf2 pathway [102]. Overall, while the results of increasing Nrf2 signaling in AD mouse models are promising, it is not clear whether they will translate to humans.

## 8. Concluding Remarks

Growing evidence indicates that AD is a multifactorial disease. Therefore, any efficacious therapy might need to target multiple proteins/factors [103]. In this regard, in addition to the classical hallmarks of the disease (i.e., Aβ and tau accumulation), it is well established that inflammation and oxidative damage are invariable features associated with the onset and progression of AD. While enormous progress has been made toward the development of anti-Aβ therapies, less progress has been made with therapies targeting inflammation and oxidative stress. Nrf2 acts as a master regulator of the cellular redox homeostasis and inflammatory response and its function is decreased in AD. Thus, not surprisingly, different approaches aimed at increasing Nrf2 function have been tried in animal models of AD, most of which have yielded successful results. Currently, several ongoing clinical trials are testing small drugs to activate Nrf2 in AD (e.g., NCT02292238; NCT02711683; NCT02085265; NCT04213391, Table 1) and some preliminary positive results are coming online [104]. Taken together, these data suggest cautious optimism for the beneficial effects of therapeutic strategies aimed at increasing Nrf2 function in AD.

## Figures and Tables

**Table 1:** Major clinical trials involving Nrf2 in AD and aging. Data from ClinicalTrials.gov, accessed on 1 March 2023.

Number ClinicalTrials.gov Identifier	Title	Study Type	Intervention/Treatment	Conditions	Number of People Enrolled	Data/Status
NCT02711683	DL-3-n-butylphthalide Treatment in Patients With Mild to Moderate Alzheimer’s Disease Already Receiving Donepezil: A Multi Center, Prospective Cohort Stud	Observational	Drug: DL-3-n-butylphthalide; Drug: Donepezil	Alzheimer’s Disease	92	2019-12 (completed)
NCT02292238	Benfotiamine in Alzheimer’s Disease: A Pilot Study	Interventional	Drug: Benfotiamine		71	2020-09 (completed)
NCT03289143	A Phase II, Multicenter, Randomized, Double-Blind, Placebo-Controlled, Parallel-Group, Efficacy, and Safety Study of MTAU9937A in Patients With Prodromal to Mild Alzheimer’s Disease		Drug: Semorinemab; Drug: Placebo ; Drug: [18F]GTP1		457	2021-01 (completed)
NCT04213391	Randomized, Double-blind, Placebo-controlled, Efficacy and Safety Study of Sulforaphane in Patients With Prodromal to Mild Alzheimer’s Disease		Dietary Supplement: sulforaphane ; Dietary Supplement: Placebo		160	2022-12 (completed)
NCT02085265	Telmisartan vs. Perindopril in Mild-Moderate Alzheimer’s Disease Patients (SARTAN-AD)		Drug: Perindopril ; Drug: Telmisartan		150	2023-09 (Recruiting)
NCT03419988	Cell Signaling and Resistance to Oxidative Stress: Effects of Aging and Exercise		Behavioral: Exercise Intervention	Aging Problems	46	2020-06 (completed)
NCT04848792	Treatment Strategy to Enhance Nrf2 Signaling in Older Adults: Combining Acute Exercise With the Phytochemical Sulforaphane		Dietary Supplement: Sulforaphane ; Dietary Supplement: Placebo capsules		30	2024-06 (Recruiting)
